# A review of *Cystoderma* (Agaricales/Basidiomycota) from China with four new species and two new records

**DOI:** 10.1080/21501203.2021.2013969

**Published:** 2021-12-22

**Authors:** Li Jia-Xin, He Mao-Qiang, Zhao Rui-Lin

**Affiliations:** a State Key Laboratory of Mycology, Institute of Microbiology, Chinese Academy of Sciences, Beijing, People’s Republic of China; b College of Life Sciences, University of Chinese Academy of Sciences, Beijing, People’s Republic of China

**Keywords:** Agaricales, multigene, phylogeny, taxonomy

## Abstract

*Cystoderma* comprises the species with heavily universal veil remnants on basidiomes, weakly to strongly amyloid basidiospores, evanescent floccose-scaly ring zone or persistent membranous ring, which were often encountered in forests and grassland. However, they were less studied than other mushroom groups mainly because of its unclearly phylogenetic position. In this study, we gathered 16 specimens from Southwest and Northwest China, where were the richest biodiversity areas in China, and produced their ITS and nrLSU sequences. The related morphological examinations and molecular phylogenetic analysis showed they belonged to eight species, of which four were new species, and named as *Cystoderma lilaceum, C. pseudoamianthinum, C. rugosolateritium, C. subglobisporum*, and of which two were new records from China, and they were *C. granosum, C. subvinaceum*. New species and new records were described in details and discussed with other species. This study not only showed the novel geographical distributions as well as high species diversity of *Cystoderma* in China, but also provided more research data for the further studies in mushrooms systematics.

## Introduction

The genus *Cystoderma* Fayod typified by *C. amianthinum* (Scop.) Fayod was established in 1889 (Fayod 1889), which belongs to Agaricales. Most species of this genus were saprotrophic and often gregariously growing on mosses, litters, or rotten wood under coniferous trees. *Cystoderma* species were often reported from temperate region (Smith and Singer 1945; Heinemann and Thoen 1977; Saar 2003; Saar and Laessoe 2006). Up to now there were 27 species of *Cystoderma* have been reported from worldwide.

*Cystoderma* species were morphologically distinguished by basidiomes usually with heavily universal veil remnants, with evanescent floccose-scaly ring zone or well-developed persistent membranous ring and weakly to strongly amyloid basidiospores (Saar 2012). In the field, *Cystodemella* was the most similar genus to *Cystoderma* in morphology as they were sharing the small to medium sized basidiomes, pileus sometimes with radially wrinkles, granulose to finely scales, and margin usually with veil remnants. Further identifications need micromorphological characteristics and molecular analyses. *Cystodermella* was characterised by inamyloid basidiospores, an evanescent floccose-scaly ring zone and presence of cheilocystidia and pleurocystidia (Saar 2012).

Historically, this genus was first placed in the tribe *Lepiota* of *Agaricus* by Fries (1821). Later, Smith and Singer raised it as a morphologically distinct genus and assigned related taxa into two sections: *Granulosa* (with inamyloid basidiospores) and *Amianthina* (with amyloid basidiospores) (Smith and Singer 1945). In 1962, Singer replaced the name of section *Amianthina* by section *Cystoderma* (Singer 1962). The first molecular study which related *Cystoderma* was published in 2002, which focused on the phylogenetic study of Agaricales (Moncalvo et al. 2002). In this study, three *Cystoderma* speices were involved, and the results showed this genus was not monophyletic. Later, Harmaja considered the results of the phylogenetic analyses in Moncalvo et al. (2002) and divided *Cystoderma* into two genera, one was kept the name of *Cystoderma* and another was named as *Cystodermella* (Harmaja 2002; Moncalvo et al. 2002). Presently the belonging of *Cystoderma* was still not clearly yet. Based on morphological characteristics, Singer (1986) proposed *Cystoderma* as a member of Tribus *Cystodermateae* of family Agaricaceae, which was characterised by free lamellae. However, because none of the species of *Cystoderma* has free lamellae, some researchers thought it should be placed in family Tricholomataceae, which was with attached lamellae (Heinemann and Thoen 1977; Kühner 1980; Bas 1988). In a molecular phylogenetic study of Agaricales, *Cystoderma* clustered together with *Cyathus* and *Crucibulum*, as a moderately supported sister clade of Agaricaceae in the Agaricoid clade (Matheny et al. 2006). Later, Garnica et al. (2007) found *Phaeolepiota* had a closer position to *Cystoderma*, then, they both clustered with *Crucibulum* and *Cyathus* in a well-supported lineage. Unfortunately, both studies only used the type species of *Cystoderma*. Based on such disagreement, in the most recently outline of Basidiomycota, *Cystoderma* was placed as incertae sedis in Agaricales (He et al. 2019).

In China, four species of *Cystoderma* have been reported, *C. amianthinum* (Scop.) Fayod, *C. carcharias* (Pers.) Fayod., and *C. aureum* (Matt.) Kühner & Romagn., *C. japonicum* Thoen & Hongo. (Tai 1979; Mao 2000; Li et al. 2015; Liu et al. 2021). In this study, together with 16 specimens collected from China, we made a comprehensive phylogenetic study of *Cystoderma* with the referenced molecular sequences from worldwide specimens (Saar et al. 2016; Saar et al. 2009; Saar 2016). Based on the phylogenetic and morphological analyses, we introduced four new species and two new recorded species from China in this paper.

## Materials and methods

### Morphological character examination

Specimens were collected in the field after taking photographs. Odour and colour changes on bruising were recorded at the same time. To avoid mixing or crushing, aluminium foil was used for wrapping. Macro-morphological features and chemical reactions of fresh specimens were recorded as soon as possible after returning from the field. Specimens were dried completely with a food drier under the temperature of 50°C, sealed in plastic bags, and deposited in the Herbarium Mycologicum Academiae Sinicae, Beijing, China (HMAS).

Anatomical and cytological features including lamellae, pileipellis, veil remnants, basidiospores, basidia and cystidia were observed. Dried specimens were examined following the protocols of Largent (Largent et al. 1986). Melzer’s reagent and 5% KOH are used for staining reaction. More than 20 measurements of microscopic features (spores, basidia and cystidia) were recorded, which include x, the mean of length by width ±SD; Q, the quotient of basidiospore length to width, and Qm, the mean of Q-values ±SD (Largent et al. 1986).

### DNA extraction and PCR

DNA was extracted from dried specimens using a Broad-spectrum plant Rapid Genomic DNA Kit (Biomed) according to the manufacturer protocol. Primers ITS4 and ITS5 were used for internal transcribed spacer (ITS), LROR and LR5 for large ribosomal subunit (nrLSU) PCR reactions (Moncalvo et al. 2000). The PCR programmesare followed Li et al. (2019). PCR products were sent to a Biomed Biotechnology commercial company for sequencing.

### Phylogenetic analyses

Sequences from Chinese specimens and NCBI GenBank database (Zhao et al. 2007; Saar 2012; Saar et al. 2016;) were used in phylogenetic analyses, and the related GenBank accession numbers were listed in Table 1. Sequences of ITS and nrLSU were aligned by Muscle version 3.6 (Edgar 2004) separately, then manually adjusted to remove ambiguous regions in BioEdit version 7.0.4 (Hall 2007). Maximum likelihood (ML) analysis was performed by RAxmlGUI 1.3 (Silvestro and Michalak 2012) under a GTRGAMMA model with one thousand rapid bootstrap (BS) replicates. Bayesian Inference (BI) analysis was performed by MrBayes v3.2.6 (Ronquist and Huelsenbeck 2003). Six Markov chains were run for 2,000,000 generations and trees were sampled every 100th generation. Burn-ins was determined in Tracer version 1.6 with an ESS value higher than 200 and the remaining trees were used to calculate Bayesian posterior probabilities (PP). The trees were displayed in Fig Tree version 1.4.0.

**Table 1.** Specimens of *Cystoderma* used in this study: sequences generated from this study are shown in bold face.

Species	Collection/voucher Number	Location	GB accession numbers/UNITE code	
			ITS	nrLSU
*Cystoderma amianthinum* epitype	TU101287	Estonia	AM946480	AM946424
*C.amianthinum*	TU101916	Newfoundland,Canada	UDB016215	-
***C. amianthinum***	**HMAS291341**	**Heilongjiang, China**	**MW242929**	-
*C. aureum*	TAAM146976	Estonia	AM946522	-
*C. aureum*	C27851	Denmark	AM946523	AM946459
***C. aureum***	**HMAS255933**	**Yunnan, China**	**MZ424458**	**MZ413916**
*C. carcharias var.carcharias* epitype	TAAM172011	Sweden	AM946483	AM946428
*C. carcharias var.carcharias*	TU106011	Estonia	UDB015074	-
*C. carpaticum* holotype	IB19750290	Poland	LT592276	-
*C. carpaticum*	CNF1/7034	Croatia	LT592274	LT592277
*C. chocoanum* isotype	NY00775586	Colombia	-	U85302
*C. chocoanum*	SP393641	São Paulo, Brazil	-	EU727143
*C. japonicum* holotype	BR5020079022647	Japan	AM946491	AM946435
*C. japonicum*	TU101697	Estonia	UDB011137	LT592278
*C. jasonis*	TU101948	Newfoundland,Canada	UDB016440	-
*C. jasonis*	TU118180	Estonia	UDB015579	-
*C. simulatum*	PDD83705	New Zealand	AM946490	AM946434
*C. simulatum*	PDD75555	New Zealand	AM946489	AM946432
*C. subvinaceum*	WU19742	Austria	AM946501	AM946441
*C. subvinaceum*	WU10567	Austria	AM946502	-
***C. subvinaceum***	**HMAS291342**	**InnerMongolia, China**	**MW242924**	**MW242941**
*C. superbum*	BR22288-75	Belgium	AM946504	AM946442
*C. superbum*	REG (Oct 1976)	Germany	AM946503	AM946443
*C. tricholomoides* holotype	BR5020125408845	Germany	UDB011633	-
*C. tricholomoides*	BR De Meyer 597	The Netherlands	UDB011634	-
*C. tuomikoskii* holotype	H6026179	Finland	AM946505	AM946444
*C. tuomikoskii*	O153775	Norway	AM946507	-
*C. andinum* isotype	C57998	Southern America	AM946481	AM946425
*C. andinum*	C58476	Southern America	AM946482	AM946426
***C. pseudoamianthinum***	**HMAS291343**	**Heilongjiang, China**	**MW242928**	**MW242940**
***C. pseudoamianthinum***	**HMAS291344**	**Heilongjiang, China**	**MW242927**	**MW242939**
***C. pseudoamianthinum***	**HMAS291345**	**Heilongjiang, China**	**MW242926**	**MW242938**
***C. pseudoamianthinum* holotype**	**HMAS255932**	**Yunnan, China**	**MZ424460**	**MZ413918**
***C. pseudoamianthinum***	**HMAS255935**	**Heilongjiang, China**	**MZ424461**	**MZ413919**
***C. granosum***	**HMAS291346**	**Gansu, China**	**MW242933**	**MW242945**
***C. granosum***	**HMAS291347**	**Gansu, China**	**MW242931**	**MW242943**
***C. granosum***	**HMAS291348**	**Gansu, China**	**MW242932**	**MW242944**
***C. granosum***	**HMAS255934**	**Gansu, China**	**MZ424459**	**MZ413917**
***C. lilaceum* holotype**	**HMAS291349**	**Gansu, China**	**MW242922**	**MW242948**
***C***. ***lilaceum***	**HMAS291350**	**Gansu, China**	**MW242923**	**MW242946**
***C. subglobisporum* holotype**	**HMAS281432**	**Tibet, China**	**MW242934**	**MW242947**
***C. rugosolateritium* holotype**	**HMAS291351**	**Gansu, China**	**MW242925**	**MW242937**
*Crucibulum leave*	CBS168.37	Sweden	MH855872	MH867377
*Crucibulum leave*	SWFC21261	Ningxia, China	DQ463357	

## Results

**Figure 1.**
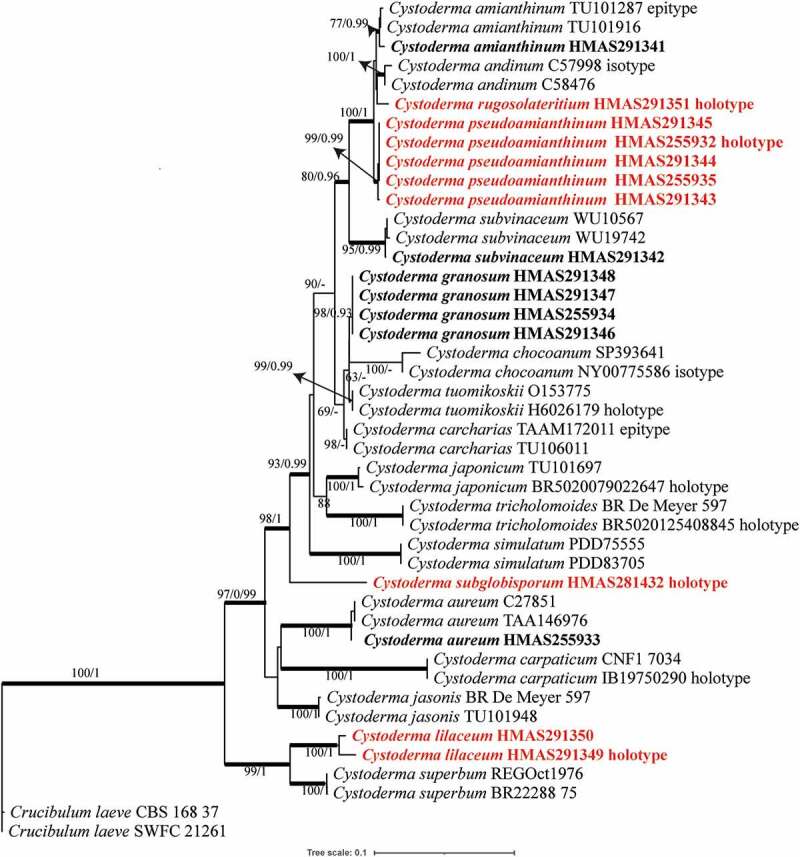
Maximum likelihood (ML) tree of *Cystoderma* based on ITS and nrLSU sequences data, rooted with *Crucibulum leave*. The bootstrap values and Bayesian posterior probabilities more than 50%/0.9 (BS/PP) are indicated at the nodes. The branches in bold mean the related PP > 0.95.

There were 44 specimens from 21 *Cystoderma* species were included in the phylogenetic analyses with an outgroup species *Crucibulum leave*. Thirty-one sequences were newly generated in this study including sixteen ITS and fifteen nrLSU sequences, which were from sixteen specimens from Northwest and Southwest China. The final alignment comprised a total of 1539 base pairs (bp) including 648 bp of ITS and 891 bp of nrLSU. The phylogenetic tree of ML and MrBaye topology were same based on ITS and nrLSU data. The Maximum likelihood tree was showed in Figure 1 with bootstrap values and Bayesian posterior probabilities indicated on the branches.

In the phylogenetic tree (Figure 1), *Cystoderma* was monophyletic with fully supported by bootstrap and PP values. Two main clades were recognised, and the basal clade comprised by the new species *C. lilaceum* and known species *C. superbum*, another was comprised by the rest sixteen species. Both clades were well supported with BS/PP values of 99/1.0 and 97/0.99 respectively. Among them, *C. amianthinum, C. andinum, C. rugosolateritium, C. pseudoamianthinum* cluster together with full bootstrap and Bayesian posterior probability values (100/1). The specimen HMAS281432 represent *C. subglobisporum* form a distinct lineage embedded in the phylogenetic tree.

## Taxonomy

### New species

**Figure 2.**
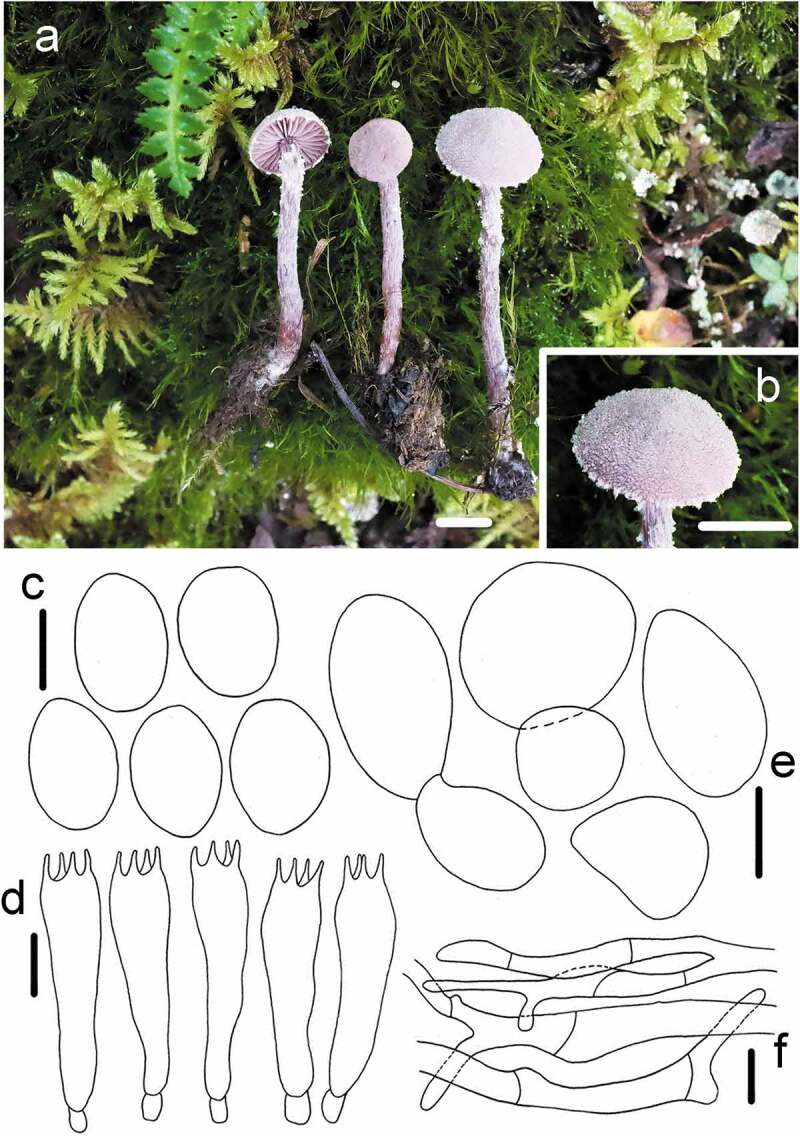
*Cystoderma lilaceum* (HMAS291349, holotype). (a–b) basidiomes in field; c. basidiospore; d. basidia; e. sphaerocysts from pileus; f. ring zone hyphae; bar a–b = 1 cm, c–d = 5 μm, e = 20 μm, f = 5 μm.

1. *Cystoderma lilaceum* R.L. Zhao, M.Q. He & J.X. Li, *sp. nov*. Figure 2

Fungal Names: FN570856

Etymology: referred to the lilac colour of the basidioma.

Holotype: CHINA, Gansu Province, Zhangye County, Qilian Mountain National Nature Reserve, 28 August 2016. *ZRL20161878* (HMAS 291349)

Macroscopic description: Pileus 5–14 mm in diam, at first conical to convex and finally expanded to applanate, surface dry, granulose, off-white to purple grey, finely covered with fibrillose sometimes, margin at first slightly decurved and appendiculate with white to greyish veil remnants. Lamellae adnexed or adnate, with 1–2 lamellulae, less than 2 mm broad, violent to pale purple, subdistant, at times somewhat ventricose. Stipe 20–35 × 2–3 mm, cylindrical, violent, a purple tinge, with evanescent floccose-scaly ring zone, above the ring zone concolorous with the lamellae, silky striate, below the ring zone densely squamulose, covered with evanescent floccose, concolorous with the pileus, the base slightly swollen. Context up to 2 mm, pale purple. Odour not distinctive.

KOH reaction: strongly black on pileus of dry specimen.

Microscopic description: *Basidiospores* 3.1–4.3 × 2.2–3 μm, [x = 3.7 ± 0.3 × 2.6 ± 0.2, Q = 1.2–1.6, Qm = 1.4 ± 0.1, n = 22], ellipsoid to narrow ovoid, smooth, hyaline, no germ pore, strongly amyloid. Basidia (11 –)14.1–21.6 × 4.3–6.4 μm, clavate, smooth, hyaline, 4-spored. *Pleurocystidia* and *Cheilocystidia* absent. *Pileipellis* composed of chains of numerous brownish sphaerocytes, globose to ellipsoid, sometimes oblong, 22.9–40 × 10.6–24.2 μm, darking in KOH. Ring zone composed of hyaline hyphae up to 5 mm in diam., branched and interwoven, attached sphaerocytes 3–4.6 × 1.5–3 μm.

Habit: Gregarious on coniferous or moss, usually in a humid environment.

Known distribution: Northwest China.

Other material examined: CHINA, Gansu Province, Zhangye County, Qilian Mountain National Nature Reserve, 31 August 2016, *ZRL20162088* (HMAS291350);

Notes: *Cystoderma lilaceum* is easily distinguished by its purple-grey caps and violent to purple gills, with a pale purple tinge overall. Compared with *C. superbum*, which is the mostly similar species with this new speceis because of both sharing with purplish basidiomes, moreover, *C. superbum* is sister to this new species with strong value (99/1) in the phylogenetic analyses. However, *C. superbum* differs by the bigger basidiomes (pileus 20–55 mm), and larger basidiospores 4.0–5.0 (– 5.5) × (2.5 –) 3.0–3.5 μm, weakly to grey amyloid. *Cystoderma haematites*, another purplish species of *Cystoderma ha*s the larger basidiospores (Huijsman 1956; Saar 2003).

**Figure 3.**
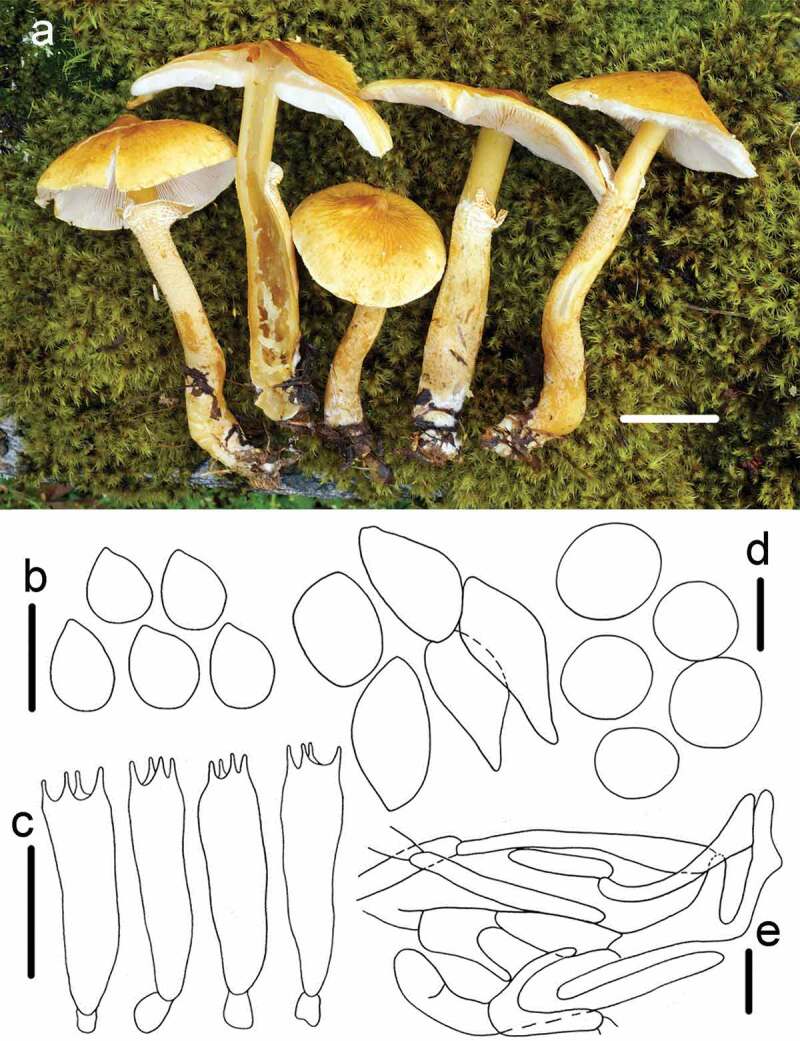
*Cystoderma subglobisporum* (HMAS281432, holotype). a basidiomes in field; b. basidiospores; c. basidia; d. sphaerocysts from pileus; e. annulus hyphae; bar a = 2 cm, b = 5 μm, c = 10 μm, d = 20 μm, e = 5 μm.

2.*Cystoderma subglobisporum* R.L. Zhao, M.Q. He & J.X. Li, *sp. nov*. Figure 3

Fungal Name: FN570857

Etymology: referred to its subglobose basidiospores.

Holotype: CHINA, Tibet Autonomous Region, Dingjie County, Chentang town, 12 September 2015, *ZRL20152031* (HMAS281432)

Macroscopic description: Pileus 43–75 mm in diam., at first convex to obstusely umbonate, then becoming campanulate broadly plane, with a distinct papilla central, surface dry, yellowish buff or yellowish-ochre, pale yellow at the edge, darker towards the disc, usually radially acute rugose, margin entire and slightly curved when young. Lamellae sinuate or sinuately adnexed, whitish, crowded, with 1–2 lamellulae of different lengths, up to 4 mm broad. Stipe 60–105 × 7–16 mm, cylindrical, hollow, enlarged base often crooked and occasionally with whitish flocs, surface dry, with well-developed persistent membranous flaring ring, coated granular, pale tan and glabrous above the ring, below the annulus covered with scaly to fibriose-scales, concolorous with the pileus. Context flesh, pale yellow, thick, up to 8–10 mm, Odour not distinctive.

KOH reaction: reddish brown on pileus of dry specimen.

Microscopic description: *Basidiospores* 3.6–4.3 × 3.2–3.7 μm, [x = 3.9 ± 0.2 × 3.4 ± 0.1, Q = 1.1–1.2, Qm = 1.1 ± 0.1, n = 20], subspherical with obvious hilar appendix, smooth, hyaline, no germ pore, strongly amyloid. *Basidia* 19.2–26.9 × 5.7–7.7 μm, clavate, smooth, hyaline, 4-spored, inamyloid. *Pleurocystidia* and *Cheilocystidia* absent. *Pileipellis* composed of numerous brownish sphaerocytes, inflated, globose to subglobose, pyriform, 30.2–48.5 × 19.8–29.5 μm, smooth, hyaline, no discoloration in KOH. Annulus composed of hyaline hyphae 3–8 μm in diam. *Arthrospores* present. *Clamp connections* abundant.

Habit: solitary on a mixture of moss and fallen leaves in forest.

Known distribution: Northwest China.

Notes: *Cystoderma subglobisporum* is characterised by relatively large basidiomes (Pileus 43–75 mm, stipe 60–105 × 7–16 mm), pileus with a yellowish buff unique papilla, well-developed persistent membranous ring, subspherical basidiospores (3.9–4.3 × 3.3–3.9 μm), strongly amyloid. Compared with other six known species, which have well-developed persistent membranous ring: *C. aureum* (Matt.) Kühner & Romagn, *C. japonicum* Thoen & Hongo, *C. carcharias* (Pers.) Fayod, *C. granosum* (Morgan) A.H. Smith. & Singer, *C. tricholomoides* Heinem. & Thoen and *C. texense* Thiers, proposed new species *C subglobisporum* differed from them not only in moelecualr phylogeny, but also in morphology. For example, *C. aureum* featured by big basidiomes (pileus 7–20 cm, stipe 10–20 × 1.5–3 cm) and inamyloid larger basidiospore (10–14 × 5–6 μm). *Cystoderma japonicus* differed by yellowish ochre to orange-yellow pileus without a definite papilla, inamyloid and longer basidiospore (4–5 × 2.5–3 μm) (Saar 2012). *Cystoderma carcharias* differed by forming pale pinkish or vinaceous coloured basidiocarp, above the ring, usually with whitish and silky striate, moreover, longer basidiospore (3.5–)4 – 5(– 6) × (2.5 –)3–4 μm. *Cystoderma granosum* differed by fruiting body covered with granulose scales, and finely powdery, and stipe surface usually brownish-yellowish above the ring. *Cystoderma tricholomoides* differed by reddish brown to bright red cap, inamyloid and longer basidiospores (4.6–6 × 2.9–3.4 μm). *Cystoderma texense* could be easily separated by the present of cheilocystidia with a spear-shaped tip (Kaunert and Léonard 2011).

**Figure 4.**
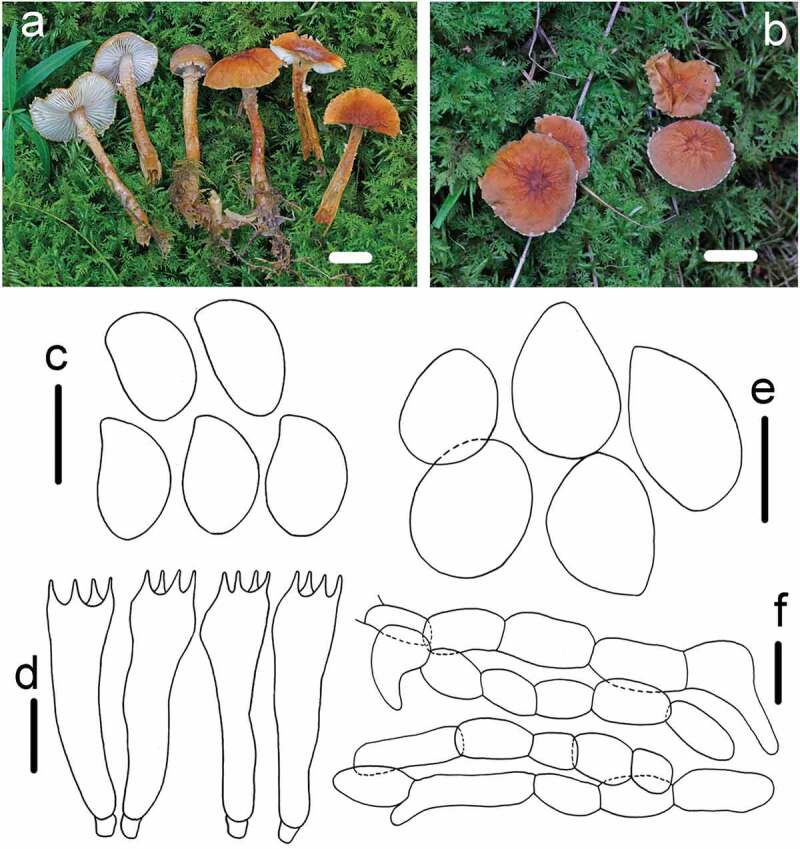
*Cystoderma rugosolateritium* (HMAS291351, holotype). (a–b) basidiomes in field; c. basidiospore; d. basidia; e. sphaerocysts from pileus; f. elements of annulus; bar a-b = 1 cm, c-d = 5 μm, e = 20 μm, f = 5 μm.

3.*Cystoderma rugosolateritium* R.L. Zhao, M.Q. He & J.X. Li, *sp. nov*. Figure 4

Fungal Name: FN570858

Etymology: referring to the brick-red colour of disc, and deep wrinkles on the pileus.

Holotype: CHINA, Gansu Province, Zhangye County, Qilian Mountains, 23 August 2017, collected by Mao-Qiang He. *QL20170293* (HMAS291351)

Macroscopic description: Pileus 10–28 mm in diam., convex to plano-convex, rusty tawny, cinnamon at the edge, turning into brick-colour towards the disc, deepen in strongly radially winkles, with darker umbo, margin decurved at first, then applanate to plane with age, appendiculate with remnants of partial veil, veil often erected, white to yellowish-brown. Lamellae creamy white to pure white, adnate to adnexed, subdistant, with 1–2 lamellulae of different lengths, ventricose, 3–5 mm broad. Stipe 38–58 × 4–8 mm, cylindrical, hollow, yellowish brown at first, changing to reddish brown when bruised or cut, with evanescent floccose-scaly ring zone, above the ring zone densely covered fribrious striate, white to yellowish. Below the ring zone, attached with small fibrillous squamules which are similar to the ring zone, scanty towards the base, Context thin, less than 2 mm broad, concolorous with stipe, Odour not distinctive.

KOH reaction: Reddish-brown on pileus of dry specimen

Microscopic description: *Basidiospores* 5.5–6.8 × 3–4.4 μm, [x = 6 ± 0.4 × 3.5 ± 0.3, Q = 1.5–2, Qm = 1.7 ± 0.1, n = 22], oblong to ellipsoid, smooth, hyaline, no germ pore, amyloid. *Basidia* 24.1–26.4 × 5.3–6.7 μm, clavate to narrowly clavate, smooth, hyaline, 4-spored, inamyloid. Pleurocystidia and Cheilocystidia absent. *Pileipellis* composed of numerous brownish sphaerocytes, 18.7–32.4 × 12–16.8 μm, oblong to ellipsoid subglobose to globose, sometimes pyriform, smooth, hyaline, no discoloration in KOH. *Annulus* composed of hyaline, filamentous hyphae, 6 μm in diam, surface layer attached cylindrical, oblong to ellipsoid sphaerocytes, 8.7–10.8 × 4.5–7.5 μm. *Arthrospores* present.

Habit: Single or scattered on moss, usually in a wet environment.

Known distribution: Northwest China.

Notes: *Cystoderma rugosolateritium* is characterised by brick-red colour of the disc, deep wrinkles on pileus, erected, whitish to yellowish brown remnants of veil, ventricose lamellae, turning reddish brown when bruised or cut. In the phylogenetic analyses, this new species represented by *QL20170293* clustered with *C. amianthinum, C. andinum* and *C. pseudoamianthinum* with full bootstrap and high Bayesian posterior probability values (100/1). Among them, *C. andinum* differed by its larger basidiospores (5.0 –)6.0–7.5(– 8.5) × (4.0 –)4.5–5.5(– 6.0) μm and pileus without wrinkles, furthermore, its brick-red on pileus fading greyish orange, retaining the darker colour at the margin for some time which could easily distinguish them in the field (Saar and Laessoe 2006). *Cystoderma amianthinum* form the yellowish buff to pale-yellow pileus, narrowly basidiospore (4.0 –)5.0–6.0(– 7.0) × (2.5 –)3.0–3.5(– 4.0), which features differed from this new species. *Cystoderma pseudoamianthinum* could be easily distinguished from this new species by the frosted powderies covered on the basidiomes, and a usually umbonate pileus (Saar and Laessoe 2006).

**Figure 5.**
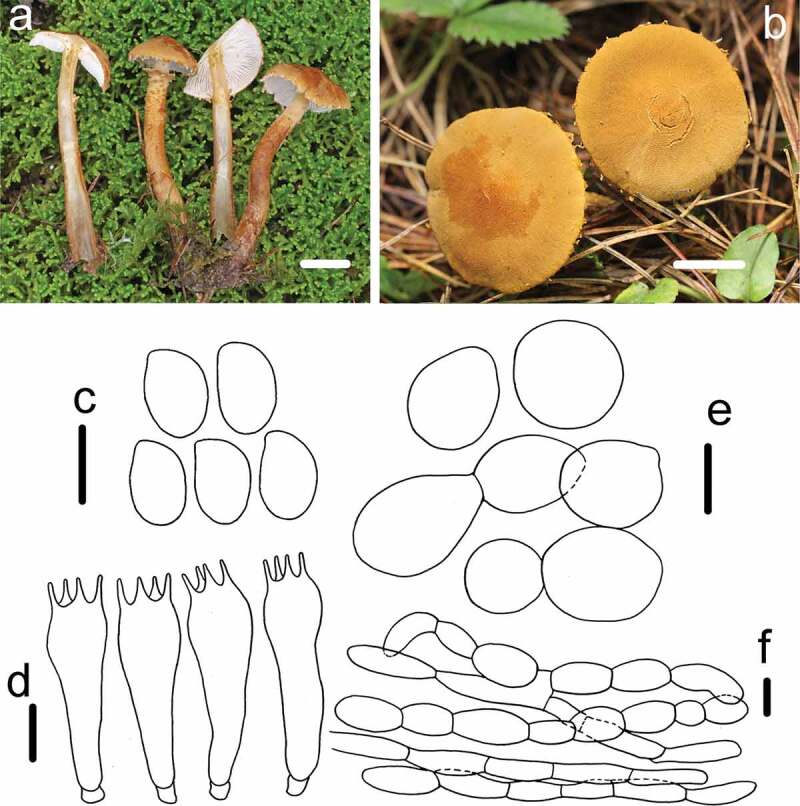
*Cystoderma pseudoamianthinum* (HMAS291345, holotype). (a–b) basidiomes in field; c. basidiospore; d. basidia; e. sphaerocysts from pileus; f. elements of annulus; bar a-b = 2 cm, a-b = 5 μm, bar c = 10 μm, bar d = 5 μm.

4.*Cystoderma pseudoamianthinum* R.L. Zhao, M.Q. He & J.X. Li, *sp. nov*. Figure 5

Fungal Name: FN570859

Etymology: referring to the basidiocarps similar to those of *Cystoderma amianthinum*

Holotype: CHINA, Yunnan Province, Zhaotong County, Dashanbao National Park, 2 August 2015, *ZRL20150996* (HMAS255932)

Macroscopic description: Pileus 24–50 mm in diam., convex to umbonate when young, becoming campanulate to slightly revolute when well developed. Margin moderately decurved at first and applanate to plane with age, usually appendiculate with remnants of partial veil, surface dry, yellow ochre at the edge, but gradually becoming yellowish brown towards the disc, densely covered with granular-furfuraceous, uneven. Lamellae adnexed, with 1–2 lamellulae of different lengths, up to 5 mm broad, white to pale cream, close to subdistant. Stipe 55–70 × 5–6 mm, cylindrical, hollow, enlarged bulbous at the base, surface dry, with evanescent floccose-scaly ring zone, white to yellowish above the ring zone, and silky striate, below the ring zone, light brown to dark brown towards the base, attached with numerous of small squamules similar to the ring zone. Context white to yellow-brown, 2–3 mm broad. Odour not distinctive.

KOH reaction: Reddish-brown on pileus of dry specimen.

Microscopic description: *Basidiospores* 5–6.6 × 3–3.9 μm, [x = 5.9 ± 0.5 × 3.4 ± 0.3, Q = 1.48–1.89, Qm = 1.74 ± 0.13, n = 32], ovoid to ellipsoid, smooth, hyaline, no germ pore, strongly amyloid. *Basidia* 19.8–23.6 × 6.4–7.6 μm, clavate, smooth, hyaline, 4-spored. *Pleurocystidia* and *Cheilocystidia absent. Pileipellis* formed by chains of numerous brownish sphaerocytes, 18.7–32.4 × 12–16.8 μm, often subglobose to globose, sometiomes pyriform or beaker-shaped, smooth, hyaline, no discoloration in KOH. *Annulus* differentiated loosely attached cylindrical, oblong to ellipsoid sphaerocytes, hyaline hyphae 5–15 in diam., branched and interwoven. *Arthrospores* present.

Habit: Gregarious or caespitose on moss under coniferous tree.

Known distribution: Northeast China and Southwest China

Other material examined: CHINA, Heilongjiang Province, Yichun County, Wuying National Forest Park, 30 August 2017, *ZRL20170815* (HMAS291345); CHINA, Heilongjiang Prov, Daxinganling Region, Wenghe National Nature Reserve, 15 August 2015, *ZRL20151656* (HMAS291343); CHINA, Heilongjiang Prov, Daxinganling Region, Bishui Forest Farm, 21 August 2017, *ZRL20170209* (HMAS291344); CHINA, Heilongjiang Province, Yichun County, Wuying National Forest Park, 30 August 2017, *ZRL20170862* (HMAS255935);

Notes: *Cystoderma pseudoamianthinum* is characterised by umbonate to campanulate pileus, pale yellow to brownish yellow from the edge to the disc, rust brown stipe and strongly amyloid basidiospores. Phylogenetic analyses showed *C. pseudoamianthinum* closed to *C. amianthinum, C. andinum* and *C. rugosolateritium* with full bootstrap and high Bayesian posterior probability values (Figure 1). However, in morphology those three speices differed from this new species. *Cystoderma andium* has brick red to fading greyish orange colour of caps and larger basidiospores (5.0 –)6.0–7.5(– 8.5) × (4.0 –)4.5–5.5(– 6.0)μm (Saar and Laessoe 2006). *Cystoderma amianthinum* featured by a yellowish buff and radially wrinkled basidiocarp, narrowly basidiospore (4.0 –)5.0–6.0(– 7.0) × (2.5 –)3.0–3.5(– 4.0), and the absent of the arthrospores in context below pileipellis. In addition, these two species had the differences of 18 positions and 4 positions in the ITS and LSU sequences respectively. The proposed another new speices from this paper, *C. rugosolateritium*, differed by brick-red colour, deep wrinkles on pilues, whitish to yellowish brown remnants of veil.

Morphologically, *C. aureum, C. japonicum, C. jasonis* and *C. muscicola* were similar to this new species because all of them were yellowish pileus, however, those two known species *C. japonicum* and *C. jasonis* have inamyloid basidiospores. (Saar 2012). Furthermore, *C. jasonis* differs smaller basidiocarp (10–25 mm), and more broadly basidiospore (5 –)6.0–7.5(– 9.0) × 3.0–4.5 (Saar 2003). *Cystoderma muscicola* (Cleland) Grgur is the most similar to this new species that both form pileus with frosted granules, stipe covered with yellowish-buff granules and a definite ring. (Grgurinovic 1997) However, this new species *C. pseudoamianthinum* usually forms annular crack on pileus which is hardly rugose, Besides, *C. muscicola* is known to be distributed in Australia (Saar 2012).

### New records for China

*Cystoderma granosum* (Morgan) A.H. Smith & Singer Papers of the Michigan Academy of Sciences 30: 120 (1945) Figure 6

**Figure 6.**
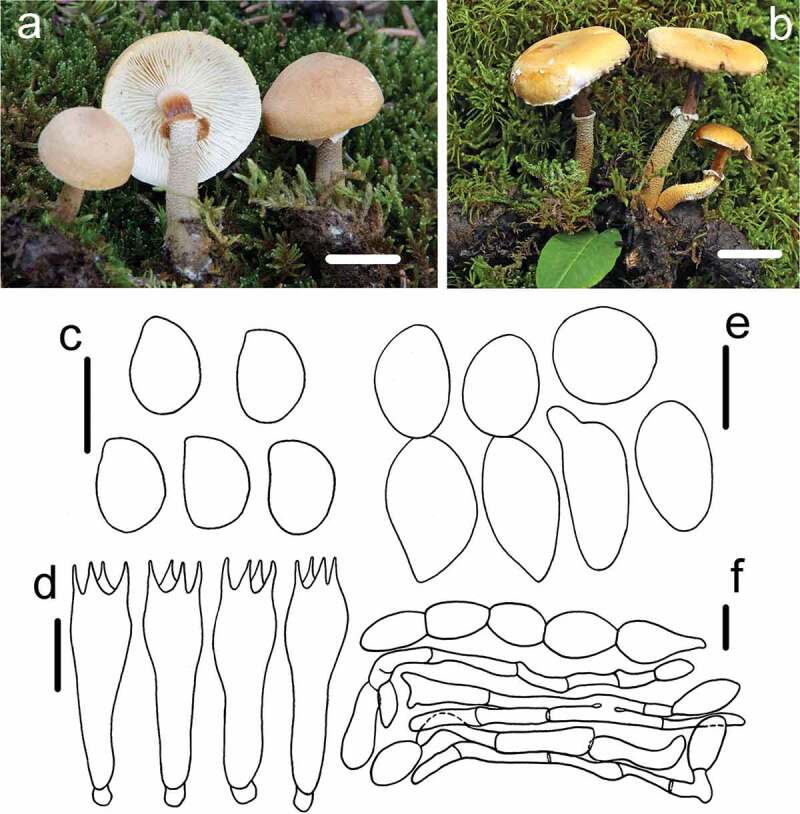
*Cystoderma granosum* (HMAS291347). (a–b) basidiomes in field; c. basidiospore; d. basidia; e. sphaerocysts from pileus; f. elements of annulus; bar a-b = 2 cm, c-d = 5 μm, e = 20 μm, f = 5 μm.

Syn: *Agaricus granosus* Morgan, Journal of the Cincinnati Society of Natural History 6: 63 (1883)

*Lepiota granosa* (Morgan) Sacc., Syll. fung. (Abellini) 5: 48 (1887)

*Mastocephalus granosu* (Morgan) Kuntze, Revis. gen. pl. (Leipzig) 2: 860 (1891)

Macroscopic description: Pileus (18–)30–50 mm in diam., convex to campanulate when young, becoming applanate to plane with age, with a conical papilla on central, surface dry, bright orange brown, greyish-brown, or yellowish ochre, finely covered granulose scales, sometimes with small finely powdery, slightly wrinkled, the margin often separates from the partial veil. Lamellae attached, whitish, with 1–2 lamellulae, close to crowded, ventricose. Stipe 30–70 × 6–12 mm, cylindrical, base enlarged, with a persistent membranous ring, coloured with the basidiocarp, above the ring, whitish to yellowish brown, below the ring, concolorous and with the same granular tissue as the cap. Odour not distinctive.

KOH reaction: Reddish-brown on pileus.

Microscopic description: *Basidiospores* 4.5–5.2 × 2.9–3.5 μm, [x = 4.9 ± 0.2 × 3.29 ± 0.1, Q = 1.4–1.5, Qm = 1.48 ± 0.04, n = 22], ellipsoid, smooth, hyaline, no germ pore, amyloid. *Basidia* 14.9–23.3 × 5.5–6.8 μm, clavate, smooth, hyaline, 4-spored, inamyloid. *Pleurocystidia* and *Cheilocystidia* absent. *Pileipellis* composed of numerous brownish sphaerocytes, inflated, subglobose to globose, sometimes with a papilla, 26.4–44.8 × 14.7–22.7 μm, smooth, hyaline, no discoloration in KOH. *Annulus* composed of hyaline hyphae 2–6 μm in diam. and oblong to ellipsoid sphaerocytes, 17.8–30.3 × 9.3–17.8 μm. *Arthrospores* present.

Habit: Gregarious or caespitose on moss, or sometimes on a mixture of pine cones and pine needle.

Known distribution: Northern America (type locality); Northwest China.

Material examined: CHINA, Gansu Province, Zhangye County, Kangle Protection station, 27 August 2018, *ZRL20181937* (HMAS291347); CHINA, Gansu Province, Zhangye County, Qilian Mountains National Nature Reserve, 23 August 2017, *QL20181937* (HMAS291346); CHINA, Gansu Province, Qilian Mountain National Nature Reserve, 1 September 2016, *ZRL20162152* (HMAS291348); CHINA, Gansu Province, Zhangye County, Qilian Mountains National Nature Reserve, 1 September 2016, *ZRL20162054* (HMAS255934);

Notes: *Cystoderma granosum* is well characterised by its large orange-brown granular basidiocarp and well-developed persistent membranous ring concolorous with the cap, the colour of stipe surface above the ring whitish at first, then becoming brownish yellow with age (Smith and Singer 1945). In the phylogenetic tree, *C. granosum* were represented by four specimens from Gansu province of China, and they clustered together with fully support. The phylogenetic tree also showed this species might close to *C. chocoanum, C. tuomikoskii* and *C. carcharias*. Compared their morphology, *C. carcharias* was the most similar species, however, it differed from *C. granosum* by forming pale pinkish or vinaceous coloured basidiocarp and the thinner stipe 30–70 × 4–8 μm (Saar 2003). Generally, characteristics of these four specimens match quite well with the original description of *C. granosum* (Smith and Singer 1945). Thus, they were identified them as *C. granosum* and provided the molecular data for this species. This species was firstly recorded for China.

*Cystoderma subvinaceum* A.H. Smith & Singer Papers of the Michigan Academy of Sciences 30: 120 (1945) Figure 7

**Figure 7.**
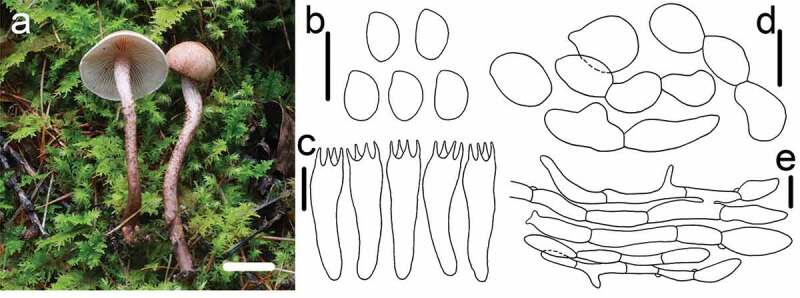
*Cystoderma subvinaceum* (HMAS291342). a. basidiomes in field; b. basidiospore; c. basidia; d. sphaerocysts from pileus; e. elements of annulus; bar a = 1 cm b-c = 5 μm, d = 20 μm, e = 5 μm.

Macroscopic description: Pileus 16–28 mm in diam., conical to campanulate, surface dry, granular, finely covered with small scales, light-pink-brown fading light greyish-brown, darken than the background, usually slightly wrinkled. Lamellae adnate to adnexed, light pink to orange white, close to crowded, Stipe 35–65 × 3–5 mm, cylindrical, with evanescent floccose-scaly ring zone, pink-brown to purple wine coloured, below the ring zone, concolorous and with the same granular tissue as the cap, gradually darken towards the base, gradually turning into tan. Odour not distinctive.

KOH reaction: clearly olive green to grey olive

Microscopic description: *Basidiospores* 3.3–4.5 × 1.9–2.8 μm, [x = 3.9 ± 0.3 × 2.5 ± 0.2, Q = 1.3–1.7, Qm = 1.5 ± 0.1, n = 22], ellipsoid, smooth, hyaline, no germ pore, amyloid. *Basidia* 15–21.4 × 4.3–5.5 μm, clavate, smooth, hyaline, 4-spored, inamyloid. Pleurocystidia and Cheilocystidia absent. *Pileipellis* composed of numerous brownish sphaerocytes, inflated, subglobose to globose, pyriform, sometiomes with a papilla, 7.8–17.9 × 19–30.1 μm, smooth, hyaline, not darking in KOH. *Annulus* composed of hyaline hyphae less than 3 μm in diam., attached short chains of large inflated sphaerocytes, 15.4–31.5 × 9.2–15 μm. *Arthrospores* present.

Habit: Caespitose on a mixture of pine needles and moss.

Known distribution: Europe (Type locality); Northern America; Northwest China.

Material examined: CHINA, Inner Mongolia Autonomous Region, Hulunbeir County, Ergun National Nature Reserve, 24 August 2017, collected by Zhi-Ling Ling, *ZRL20170498* (HMAS291342).

Notes: *Cystoderma subvinaceum* is characterised by light pink-brown to vinaceous red pileus and the stipe, KOH reaction strongly staining olive green to grey olive, present of amyloid basidiospore. (Hausknecht 1994). In the phylogenetic analysis, our specimen HMAS291342 clustered with other two specimens of *C. subvinaceum*, and formed a clade with highly supports. Then, *C. subvinaceum* was sister to *C. amianthinum, C. andinum, C. pseudoamianthinum* and *C. rugosolateritium* (Figure 1). *Cystoderma superbum* was the most phenotypically similar species to *C. subvinaceum* as both having purplish colour of basidiocarps, However, *C. superbum* has the darker colour on pileus, such as purplish red to vinaceous purple, weakly grey amyloid except for the suprahilar zone strongly amyloid, KOH reaction reddish brown (Saar 2003). *Cystoderma subvinaceum* the pileipellis colours olivaceus grey in KOH (Saar et al. 2009). For other purplish coloured species, *C. haematites* and *C. lilaceum*, the large basidiospore of *C. haematites*, or the smaller basidiospores and basidiomes of *C. lilaceum* could be used to distinguished them (Huijsman 1956). Based on the morphology and molecular phylogeny, we identifed the Chinese specimen as *C. subvinaceum* and it was the first record for China.

### Other known species from China

*Cystoderma amianthinum* (Scop.: Fr.) Fayod, Ann. Sci. Nat., Bot., Sér. VII 9: 351. 1889.

≡ *Agaricus amianthinus* Scop., Fl. carn., Ed. 2, 2: 434. 1772.

≡ *Agaricus granulosus* var. *amianthinus* (Scop.) Fr., Epicr.: 18. 1838.

≡ *Lepiota granulosa* var. *amianthina* (Scop.: Fr.) P. Kumm., Führer Pilzk.: 136. 1871.

≡ *Lepiota amianthina* (Scop.: Fr.) P. Karst., Hattsvamp.: 15. 1879.

≡ *Armillaria amianthina* (Scop.: Fr.) Kauffman, Pap. Michigan Acad. Sci. 2: 60. 1923.

Known distribution: Europe (Type locality), Africa, Northern America, Southern America, widely distributed in China

Material examined: CHINA, Heilongjiang Province, Mohe County, Lingfeng National Nature Reserve, 19 August 2017, *ZRL20170035* (HMAS291341)

Notes: *Cystoderma amianthinum* is the type species of this genus, which is characterised by a yellowish-buff pileus, white lamellae and amyloid, ellipsoid to oblong basidiospores (5–6 × 3–3.5 μm) (Saar et al. 2009). In China, *Cystoderma amianthinum* is widely distributed, and usually can be found in coniferous and broad-leaved mixed forest (Tai 1979, Mao 2000,, Li et al. 2015).

*Cystoderma carcharias* (Pers.: Fr.) Fayod, Ann. Sci. Nat., Ser. 7, 9: 351. 1889.

≡ *Agaricus carcharias* Pers., Tent. Disp. Meth. Fung. 18. 1797.

≡ *Agaricus granulosus* var. *carcharias* (Pers.) Fr., Epicr.: 18. 1838.

≡ *Lepiota granulosa* var. *carcharias* (Pers.) P. Kumm., Führer Pilzk.: 136. 1871.

≡ *Lepiota carcharias* (Pers.: Fr.) P. Karst., Hattsvampar: 14. 1879.

= *Cystoderma fallax* A.H. Sm. & Singer, Pap. Michigan Acad. Sci. 30: 116. 1945.

= *Cystoderma intermedium* Harmaja, Karstenia 19: 27. 1979.

Known distribution: Europe (Type locality), Africa, Asia-Temperate, Northern America, Southern America, Northeast China

Notes: *Cystoderma carcharias* is characterised by its pale pinkish or vinaceous coloured basidiocarp, a persistent membranous ring on stipe and amyloid, broadly ellipsoid to ellipsoid basidiospores (4–5.5 × 3–4 μm) (Saar et al. 2009). In our phylogenetic tree (Figure 1), *C. carcharias* formed a good lineage with *C. tuomikoskii, C. chocoanum and C. granosum*. This showed the same as previous studies (Saar et al. 2009; Saar 2012; Saar *et al*. 2016). In China, *C. carcharias* is distributed in Northeast China, and prefer coniferous and mixed forests, on moss and decaying litter. Mao *et al*. (2002) first recorded this species in China, also recorded another closely related species *C. fallax*, which was synonymised with *C. carcharias.*

*Cystoderma japonicum* Thoen & Hongo, Trans. Mycol. Soc. Japan 26 (1): 23 (1985)

≡*Cystodermella japonica* (Thoen & Hongo) Harmaja, Karstenia 42: 46.2002.

Known distribution: Europe, Asia-Temperate (Type locality), Sichuan China, Gansu, China.

Notes: *Cystoderma japonicum* is characterised by yellow-ochre to orange-yellow pileus, a persistent membranous ring on stipe and inamyloid ellipsoid basidiospores (4–5 × 2.5–3.5 μm) (Saar et al. 2009). Harmaja (2002) transferred *C. japonicum* to genus *Cystodermella* because possessing inamyloid basidiospores, however, in the phylogenetic analyses of ITS and nrLSU regions by Saar *et al*. (2016), showed that it belongs to genus *Cystoderma*, besides, *C. japonicum* and *C. tricholomoides* formed a well-supported sister group, this showed the same in our phylogenetic tree (Figure 1). In China, *C. japonicum* is recorded by Yuan *et al*. (2013) in Sichuan and Gansu Province, gregarious on coniferous forest and broad-leaved forest.

*Cystoderma aureum* (Matt.) Kühner & Romagn., Fl. Analyt. Champ. Supér. (Paris): 393 (1953)

= *Phaeolepiota aurea* (Matt.) Maire, Icones selectae Fungorum, 6 Texte general 6: 111 (1928)

Known distribution: Europe (Type locality), Asia-Temperate, Northern America, Southwest China

Material examined: CHINA, Yunnan Province, Xianggelila County, Napahai Nature Reserve, 8 August 2014, *ZRL2014489* (HMAS255933)

Notes: *Cystoderma aureum* is easily distinguished by possessing inamyloid basidiospores, and having a persistent membranous ring on upper part of the stipe. (Saar 2012) Besides, our examined specimens HMAS255933 producing larger basidiomos than other species of this genus. (Pileus 30–45 mm in diam; Stipe 10.5–12.5 × 1.2–1.5 cm). Since the type sequences was not obtained, scientific name *C. aureum* or *Phaeolepiota aurea* has always been controversial. Liu et al. (2021) did a research of Squamanitaceae, three *Cystoderma* species and *P. aurea* clustered together, they thought *P. aurea* and *C. superbum* was not well accommodated this genus because inamyloid fusoid and asperulate basidiospores and a small area of the basidiospore amyloid, however, only four species involved. Saar (2016) supported the inclusion of *Phaeolepiota* in genus *Cystoderma*, they perferred *Cystoderma aureum* with more species based on ITS and nrLSU sequence data. In this study, we used 18 *Cystoderma* species based on ITS and nrLSU sequence data, the specimen HMAS255933 clusted in *Cystoderma aureum* and nested within *Cystoderma*, therefore, we continue to recognise *Cystoderma aureum*.

## Discussion

The genus *Cystoderma* embraces 30 species in the world, and sequences from 19 species of this genus are available in the public sequence repositories presently. (Saar et al. 2009; Saar 2012; Saar et al. 2016). Based on a combination of morphological and phylogenetic analyses, 16 specimens of *Cystoderma* from Northwest and Northeast China were identified as eight species including of four new species *C. lilaceum, C. pseudoamianthinum, C. rugosolateritium, C. subglobisporum*, two new records *C. granosum, C. subvinaceum* and four known species *C. amianthinum, C. carcharias, C. japonicum* and *C. aureum*. Currently, and so that there are eleven species recorded in China, and they are distributed in Yunnan, Sichuan, Gansu, Inner Mongolia, Heilongjiang provinces and Tibet which is a new distribution of *Cystoderma*. In most cases, they can be found in forest ecosystems, grassland, mosses, litter and rotten wood under coniferous forest and broad-leaved forest. Therefore, *Cystoderma* has the widely distribution in China and could adapt variable habitats too.

*Cystoderma* and *Cystodermella* the two genera are most similar in morphology in the field, such as small to medium-sized basidiomes, pileus covered radially wrinkled and often granulose to finely scales. However, most *Cystoderma* species have weakly to strongly amyloid basidiospores, sometimes with a persistent membranous ring and none cheilocystidia and pleurocystidia, while *Cystodermella* has inamyloid basidiospores, evanescent floccose-scaly ring zone and often having cheilocystidia and pleurocystidia. Among *Cystoderma*, the morphological characters of different species are also variable, for example *C. aureum, C. japonicum, C. texense, C. tricholomodies*, total those four species have inamyloid basidiospores which is different from the rest *Cystoderma* species. Besides, there are six species in total constitute membranous ring, *C. subglobisporeum, C. japonicum, C. aureum, C. carcharias, C. granosum, C. tricholomodies*, while the rest species only present a floccose-scaly ring zone. Generally, the accurate delimation of each species in this genus need to combine those different morphological features and molecular phylogenetic analysis.

## Supplementary note

In the paper “He M.Q., Hyde D.K., Cheewangkoon R., Zhao R.L. (2020) Two new species of *Micropsalliota* (Agaricaceae/Agaricales) from Thailand, Phytotaxa 453(2):137–144”, the authors propose corrections for abbreviation of herbarium where holotypes were deposited, which incorrectly published due to typographical errors in the papers, as follows:

*Micropsalliota albella* M.Q. He & R.L. Zhao (Holotype: MFLUCC17-1342) is to be corrected to (Holotype: MFLU17-1342).

*Micropsalliota purpureobrunneola* M.Q. He & R.L. Zhao (Holotype: MFLUCC17-1343) is to be corrected to (Holotype: MFLU17-1343).
